# Estimating maternal mortality: what have we learned from 16 years of surveys in Afghanistan?

**DOI:** 10.1136/bmjgh-2019-002126

**Published:** 2020-05-03

**Authors:** Sandra Alba, Egbert Sondorp, Elisabeth Kleipool, Rajpal Singh Yadav, Arab S Rahim, Konrad T Juszkiewicz, Gilbert Burnham

**Affiliations:** 1 KIT Royal Tropical Institute, Amsterdam, The Netherlands; 2 Johns Hopkins University Bloomberg School of Public Health, Baltimore, Maryland, USA

**Keywords:** epidemiology, health systems evaluation, indices of health and disease and standardisation of rates, maternal health, medical demography

**Summary box**

- Maternal mortality is notoriously difficult to measure because even in high-mortality areas maternal deaths are relatively rare. These difficulties are further compounded in low-income and conflict-affected settings such as Afghanistan.
- Between 2002 and 2018, maternal mortality estimates for Afghanistan have been provided by six surveys, using a variety of costly methodologies yet yielding contradictory and sometimes implausible results.
- The ‘failure’ of household surveys to provide robust data on maternal mortality in Afghanistan is a call to reconsider the value of maternal mortality measurements to assess safe motherhood interventions in low-income and conflict-affected settings.
- We encourage stakeholders involved in the commissioning and use of maternal mortality estimates in Afghanistan and similar contexts to take stock of experiences so far and carefully consider how to best make use of existing resources.
- In the short-term efforts to measure improvements in maternal health should be redirected towards measurements of access, use and quality of services for pregnant women and women giving birth, with longer-term investments towards civil registration as a source of robust maternal mortality data.

## Introduction

‘Particularly hard hit by Afghanistan's 23 years of war, civil strife and Taliban misrule are Afghan women, who are experiencing what health officials call ''catastrophic'' death rates associated with pregnancy and childbirth’.^1^ The opening paragraph of this 2002 *New York Times* article captures how women’s health became, and still is, a cornerstone of development aid in Afghanistan. As a result, maternal health measurements have become an important tool for ‘evidence-based advocacy’,^2^ as in many other countries grappling with poor maternal health. Maternal mortality estimates in particular, have played a major role in justifying external assistance to the Afghan healthcare system^1^ and in documenting maternal health improvements as a legacy of the 2001 intervention and successive foreign involvement in the country.^3^

Sixteen years later, we released the results of the Afghanistan Health Survey (AHS) 2018.^4^ It was the second nationally representative survey conducted within the frame of System Enhancement for Health Action in Transition (SEHAT),^5^ a service delivery and health systems strengthening project implemented between 2015 and 2018. SEHAT was managed by the Afghan Ministry of Public Health and financed by the World Bank-administered Afghanistan Reconstruction Trust Fund, with the World Bank, European Union, USA and Canada as major donors. KIT Royal Tropical Institute, based in the Netherlands, was selected as the third-party monitor for SEHAT and was responsible for the implementation of two AHSs in 2015 and 2018. The SEHAT monitoring and evaluation framework had a strong focus on maternal and child health^5^ and included maternal mortality. Despite considerable efforts to measure maternal mortality in both surveys, we did not include any estimates in either of the final reports due to concerns about their validity.

In this commentary, we contextualise our experience within the history of previous efforts to measure maternal mortality in Afghanistan. We review our own lessons learnt and reflect, more globally, on their implications. We argue that our Afghanistan case-specific experience exemplifies the well-known shortcomings of surveys to measure maternal mortality and should act as a call to reconsider its value in assessing safe motherhood interventions in Afghanistan and other low-income and conflict-affected settings.

## Historical overview

The maternal mortality ratio (MMR) is defined as the number of maternal deaths during a given time period per 100 000 live births during the same time period.^6^ Maternal mortality is notoriously difficult to measure because even in high-mortality areas maternal deaths are relatively rare. Challenges broadly fall into two categories^7^: (1) problems differentiating between deaths *due* to the pregnancy as opposed to merely happening *during* the pregnancy (the latter being measured by the pregnancy-related mortality ratio and (2) problems finding deaths, particularly encountered in settings such as Afghanistan, where geography and security can severely hinder access to households.

**Figure 1:**
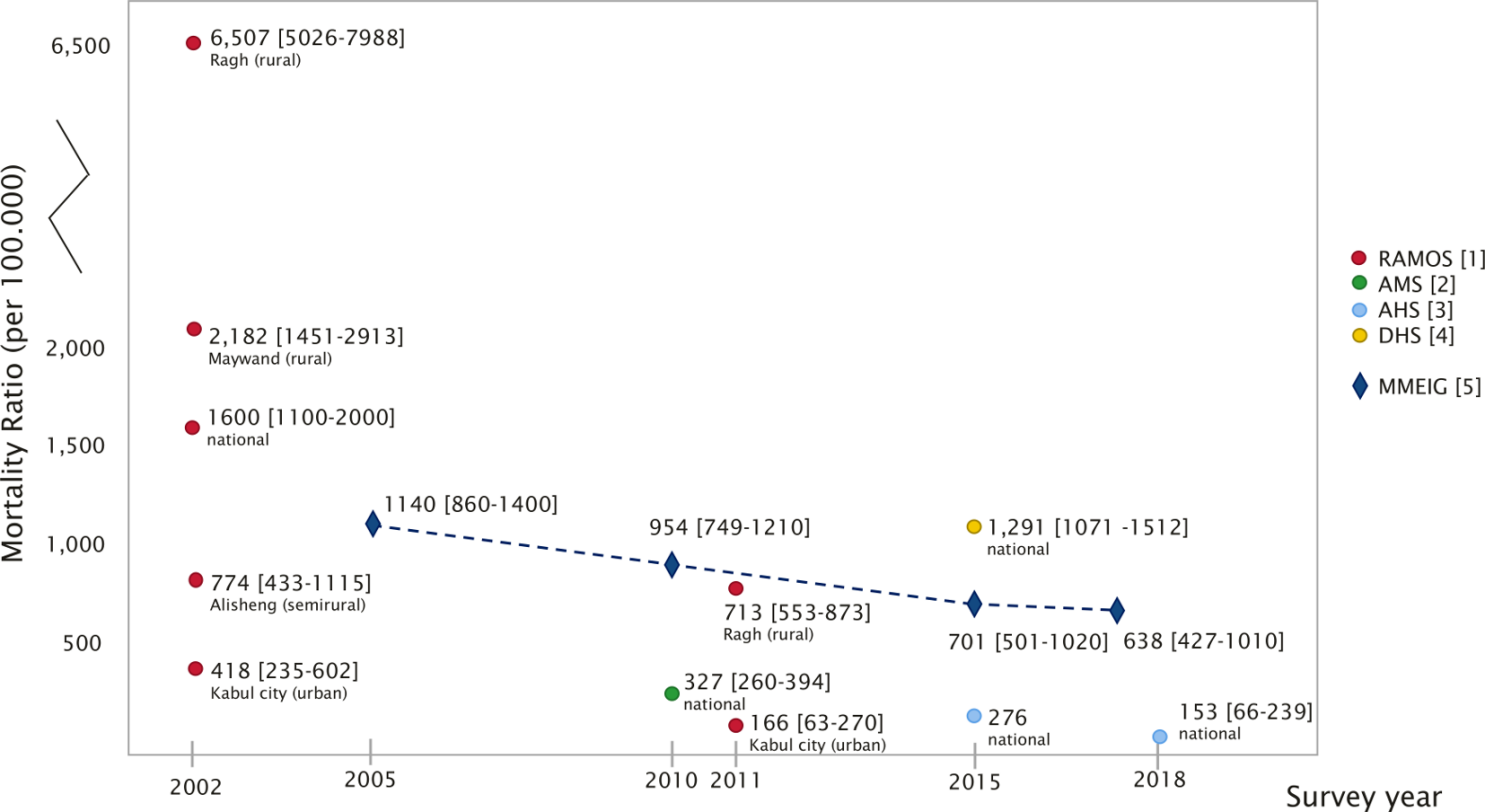
Maternal mortality estimates in Afghanistan from 2005 to 2018. (1) RAMOS, providing MMR estimates; (2) AMS, providing PRMR estimates; (3) AHS, providing MMR estimates; (4) DHS, providing PRMR estimates; (5) internationally comparable MMR estimates by the Maternal Mortality Estimation Inter-Agency Group, WHO, UNICEF, UNFPA, World Bank Group and the United Nations population division, providing MMR estimates. AHS, Afghanistan Health Survey; AMS, Afghan Mortality Survey; DHS, Demographic and Health Survey; MMR, maternal mortality ratio; PRMR, pregnancy-related mortality ratio; RAMOS, Reproductive Age Mortality Survey.

Between 2002 and 2018, maternal mortality estimates for Afghanistan have been provided by six surveys funded by external donors (primarily United States Agency for International Development and the World Bank) and implemented by or in close collaboration with international institutions (table 1 and figure 1). The surveys used a variety of costly methodologies yielding sometimes contradictory and implausible results. The estimates vary from 6507 deaths per 100 000 births in Ragh in 2002 to 153 nationally in 2018. UNICEF cut-offs^8^ help put this in perspective: MMR values above 1000 are considered extremely high and are only reported nationally in a few sub-Saharan countries, while countries neighbouring Afghanistan have low (MMR below 300) or very low (MMR below 100) maternal mortality. While a decrease in maternal mortality is in line with UN Inter-agency Group’s modelled estimates,^6^ the large variation between estimates, even between surveys conducted at the same time point (eg, 2015) is sobering.

**Table 1:** Overview of survey methodologies

Survey	Survey year	Recall period	Sampling	Method of estimation	Organisations responsible
RAMOS 1^9 10^	2002	3 years prior to the survey	Women of reproductive age (15–49 years) 13 848 households Four selected districts in four provinces: Kabul city, Kabul province (urban); Alisheng district, Laghman province (semirural); Maywand, Kandahar province (rural); and Ragh, Badakshan province (rural, most remote).	Verbal autopsies	Funding: UNICEF, CDC and USAID Implementation: CDC, UNICEF and MoPH
AMS^24^	2010	3 years prior to the survey	Women of reproductive age (12–49 years) 22 351 households Nationally representative	Verbal autopsies	Funding: USAID and UNICEF Implementation: MoPH, CSO, ICF, Indian Institute of Health Management Research and WHO/EMRO
RAMOS 2^14^	2011	3 years prior to the survey	Women of reproductive age (15–49 years) 25 043 households Urban area of Kabul city and the rural area of Ragh, Badakhshan	Verbal autopsies	Funding: USAID Implementation: Johns Hopkins Bloomberg School of Public Health and MoPH
AHS^25^	2015	3 years prior to the survey	Women of reproductive age (12–49 years) 23 118 households Nationally representative	Verbal autopsies	Funding: World Bank/MoPH Implementation: KIT Royal Tropical Institute and Silk Road Training and Research Organisation
DHS^13^	2015	7 years prior to the survey	Women of reproductive age (15–49 years) 22 351 households Nationally representative	Sisterhood method	Funding: USAID Implementation: CSO, MoPH and ICF
AHS^4^	2018	3 years prior to the survey	Women of reproductive age (15–49 years) 19 684 households Nationally representative	Verbal autopsies	Funding: World Bank/MoPH Implementation: KIT Royal Tropical Institute and CSO

AHS, Afghanistan Health Survey; AMS, Afghan Mortality Survey; CSO, Central Statistics Organisation; DHS, Demographic and Health Survey; EMRO, Eastern Mediterranean Regional Office; MoPH, Ministry of Public Health; RAMOS, Reproductive Age Mortality Survey; USAID, United States Agency for International Development.

The first MMR estimates produced for Afghanistan came from the Reproductive Age Mortality Survey (RAMOS) 1 study in 2002. The study reported a national estimate of 1600 deaths per 100 000 live births and four subnational estimates, ranging from 418 in Kabul to 6507 in Ragh.^9^,^10^ These widely quoted estimates sparked off the key focus on maternal mortality in policy and planning for Afghanistan.^1 3 11^ Set against the values, the AMS 2010 results with an MMR of 327 were criticised as implausibly low and potentially jeopardising future investments in maternal health in the country.^3 12^ On the other hand, the DHS 2015 estimates with an MMR of 1291 was criticised for being too high. Although this likely overestimation was openly acknowledged in the DHS report,^13^ this estimate is still regularly quoted as the MMR for Afghanistan^11^ though with some reservations at times.^12^ In 2011, a second RAMOS study was conducted in two areas of Afghanistan.^14^ The study showed a large decrease in the MMR, but the MMR decrease in Ragh from 6507 to 713 does cast some doubts on the validity of the RAMOS estimates in general.

Against this backdrop, the two AHS estimates (276 in 2015 and 153 in 2018) were criticized for being too low. Concerns regarding the AHS estimates were founded on anomalies in data collection. We conducted further analyses in 2018 which confirmed the likely underestimation of the AHS 2018 MMR (see online supplementary file 1). Local policy makers heavily debated the publication of these estimates in the final AHS 2015 and AHS 2018 reports and ultimately neither report included them. To the best of our knowledge, the AHS estimates are not officially used by public health authorities in Afghanistan.

10.1136/bmjgh-2019-002126.supp1 Supplementary data

Despite the wide variation in MMR estimates over time, a descending trend can be discerned. This is in line with evidence of remarkable increases in access to care and coverage of several maternal care interventions (eg, antenatal care, skilled birth attendance and births in health facilities).^4 15^ These increases are in turn reflected in decreases in the UN Inter-agency Group estimates of maternal mortality (which rely on skilled birth attendance as a predictor in their models).^6^

## Doing things right and doing the right things

After over a decade of trial and error, the time has come to review lessons learnt before further attempts to estimate maternal mortality in Afghanistan. The triple-loop learning framework^16^ offers a useful structure for these reflections. Single-loop learning consists of identifying errors (*Are we doing things right?*). Double-loop learning goes a step further and questions methodologies and analytical frameworks (*Are we doing the right things?*). Finally, triple-loop learning is deeper questioning of purpose and legitimacy (*Should we be doing anything at all?*).

### Are we doing things right?

Our review of AHS 2015 and AHS 2018 data and processes revealed that a number of deaths were probably missed. Following fears of data fabrication in hard-to-access areas in 2015, we significantly tightened quality control for theAHS 2018. This included (1) enlisting a team of external independent monitors (in addition to the internal monitors) to randomly revisit households and (2) rapid statistical review of selected data before data entry to ensure data credibility. Yet, in spite of these improvements, it appears that deaths were still under-reported in 2018. This could be due to further issues in our survey operations. Indeed, we did not use field-check tables, which could have helped to ensure internal consistency between births and related events for children and mothers. However, more profound reasons such as grief or shame may also be at play,^17^ leading to both under-reporting of all deaths or inaccurate reporting of maternal mortality as deaths from other causes.

In short, it is difficult to *‘*do things right’ when measuring maternal mortality in Afghanistan. Survey implementers should therefore carefully design, implement and describe their quality assurance mechanisms so as to enable users to contextualise the magnitude of their maternal mortality estimates.

### Are we doing the right things?

There is a reasonable consensus that surveys are not suited to measure statistically rare events such a maternal deaths.^9^ Civil registration and vital statistics (CRVS) information systems are hailed as the prime source of vital statistics,^18^ with sample surveys, censuses and modelled estimates common but imperfect alternatives. Yet Afghanistan, like many other low-income and middle-income countries, does not have adequate CRVS systems. Although the government revitalised vital events registration after the Taliban regime (in 2001) with the support of UNICEF and WHO, a recent report confirms that limited death registration takes place and causes of death are often not recorded accurately.^19^ Afghanistan set a fairly unambitious target of 20% registration by 2020,^19^ showing that it is a long way from being able to rely on CVRS-based MMR estimates. On the other hand, the dual record system, a sentinel site approach to civil registration successfully implemented in India,^20^ may hold promise for Afghanistan and could pave the way towards strengthening national civil registration and national statistics.

In other words, the failure of household surveys to provide robust data on maternal mortality confirms that they are not ‘the right thing’. However, it is not obvious what the right thing exactly is, especially in the short to medium term. Nevertheless, there is a strong case to strengthen national civil registration and national statistics in Afghanistan.

### Should we be doing anything at all?

As has already been argued since the mid-1990s, the measurement of MMR using surveys is not a good use of scarce resources.^21^ This critique is based not only on measurement difficulties described previously but also on challenges to attribute any decreases (when discernible) to specific interventions. This logic resonates particularly in conflict-affected settings such as Afghanistan, where the ethical imperative to act is arguably stronger than the necessity to measure, especially if we cannot measure well. This critique is related to calls to focus on process indicators (such as access, use and quality of healthcare services) on the causal pathway towards decreased mortality, as has been done by a number of recent studies in Afghanistan.^15 22 23^ These indicators are relatively easy to measure and more useful to inform country-level planning than the MMR, since they can also be disaggregated by equity variables.^2 12^ These technical considerations fit into broader discussions on the political drivers of maternal mortality measurement. It has been argued that the scientific practice which emphasises maternal mortality measurement is driven by global accountability needs to monitor and justify donors’ investments at the expense of governments’ local programme planning needs and accountability towards constituents, for whom data on equitable access to care would be much more relevant.^2^

In summary, diverse calls question the usefulness of the MMR to monitor safe motherhood interventions, suggesting that perhaps we ‘should not be doing anything at all’ to measure it in Afghanistan.

## Conclusion

What have we learnt from 16 years of maternal mortality surveys in Afghanistan? Not much about actual mortality levels—though contextual evidence suggests they are declining—but enough to know that survey methodologies are not providing robust MMR estimates. While the arguments we presented are not new, the Afghanistan experience exemplifies the well-known shortcomings of surveys to measure maternal mortality, particularly exacerbated in fragile and conflict-affected settings. Before further efforts to measure maternal mortality are undertaken in Afghanistan, we encourage stakeholders involved in the commissioning and use of these estimates to take stock of experiences so far and to carefully consider how to best make use of existing resources. Based on our experience, we believe the time has come to temporarily halt attempts to measure maternal mortality in Afghanistan through surveys and to explore alternative methods. In the short term, efforts to measure improvements in maternal health should be redirected towards measurements of availability, access and quality of services for pregnant women and women giving birth, with a longer-term investments towards CRVS systems as a source of robust maternal mortality data.

## Data Availability

No data are available.
