# Correction: Estimating maternal mortality: what have we learned from 16 years of surveys in Afghanistan?

**DOI:** 10.1136/bmjgh-2019-002126corr1

**Published:** 2022-02-03

Alba S, Sondorp E, Kleipool E, *et al*. Estimating maternal mortality: what have we learned from 16 years of surveys in Afghanistan? *BMJ Global Health* 2020;5:e002126. doi:10.1136/bmjgh-2019-002126

This article has been corrected since it was published online to reflect two incorrect statements. The errors were in paragraph 3 of Page 2 of the pdf version of the article and pertain to the maternal mortality ratio (MMR) cited from the two RAMOS studies^1 2^

We revised this sentence ‘While in a preliminary press release the authors disclosed a national estimate of 1600 deaths per 100, 000 live births,^9^ the survey’s official publication in 2005 only presented four subnational estimates, ranging from 418 in Kabul to 6507 in Ragh.^10^’ to:

The study reported a national estimate of 1600 deaths per 100 000 live births and four subnational estimates, ranging from 418 in Kabul to 6507 in Ragh. ^9,10^

The second incorrect statement was ‘The study showed a large decrease in the MMR, but the enormous MMR decrease especially in Ragh from 6507 in 2002 to 166 in 2011 does cast some doubts on the validity of the RAMOS estimates in general.’ It was revised to:

The study showed a large decrease in the MMR, but the MMR decrease in Ragh from 6507 to 713 does cast some doubts on the validity of the RAMOS estimates in general.

## REFERENCES

1. Bartlett LA, Mawji S, Whitehead S, *et al*. Where giving birth is a forecast of death: maternal mortality in fourdistricts of Afghanistan, 1999-2002. *Lancet* 2005;365:864–70.
2. Bartlett L, LeFevre A, Zimmerman L, *et al*. Progress and inequities in maternal mortality in Afghanistan(RAMOS-II): a retrospective observational study. *Lancet Glob Health* 2017;5:e545–55.

