# Supplementary material for: Estimating maternal mortality: what have we learned from 16 years of surveys in Afghanistan?
Source: BMJ Glob Health. 2020 May 3;5(5):e002126. doi: 10.1136/bmjgh-2019-002126 (PMC7228470; doi:10.1136/bmjgh-2019-002126)

Supplementary file 1: Internal consistency of AHS 2018 maternal mortality estimates

Taking into account both Afghanistan's historical survey estimates and the Inter Agency projections (Figure 1 in main article), maternal mortality estimates derived from the AHS 2018 appear lower than expected. The validity of the AHS estimates was assessed by comparing the various mortality and maternal health estimates in the AHS survey and assessing whether their relationships with each other are consistent with other country data. For this purpose we downloaded early child mortality, maternal mortality and maternal health indicators for the DHS StatCompiler[1] for the 158 DHS surveys conducted between 2000 and 2018 and produced a series of scatterplots for indicators known to be associated with each other.

First of all we sought reassurance that there were no issues with child mortality estimates. As can be seen from Figures 1a-1c below, there indeed do not appear to be any concerns with regards to the neo-natal, infant and under-five mortality in the AHS 2018. The relationships between these three indicators is according to what has been observed in other countries.

In terms of the maternal mortality estimates, we observed a plausible relationship between the AHS pregnancy-related mortality ratio and early childhood mortality indicators (Figures 2a-2c) as well as the maternal health indicators (Figures 3a-4c). However the pregnancy-related mortality ratio is consistently on the lowest side possible given the values of early child mortality and maternal health. There are no other countries with levels of neo-natal, infant and under-five mortality observed in Afghanistan, that have such low levels of maternal mortality; similarly, there are no countries with levels of skilled birth attendance, contraceptive prevalence rate and antenatal care observed in Afghanistan, that have such low levels of maternal mortality. In general, there are only 4 countries with lower MMR estimates since 2000 in the DHS database. We concluded from these analyses that the maternal/pregnancy-related mortality rate in the AHS 2018 has been underestimated.

Figure 1. Associations between early childhood mortality rates in AHS 2018 and DHS surveys conducted between 2000 and 2018 (N=139)

(a) Neonatal mortality and infant mortality

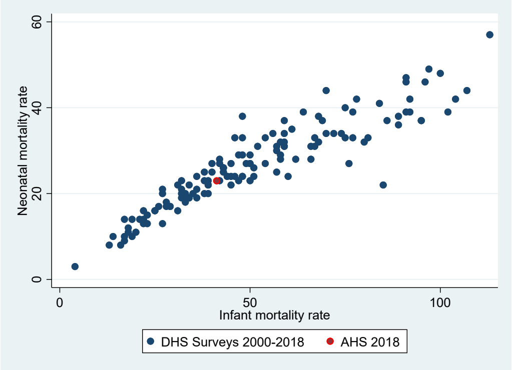

(b) Neonatal mortality and under-five mortality

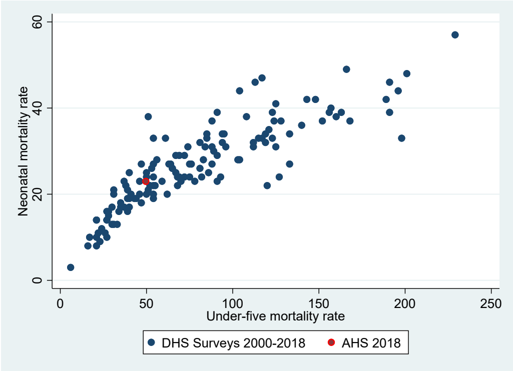

(c) Infant and under-five mortality

[1] https://www.statcompiler.com/en/

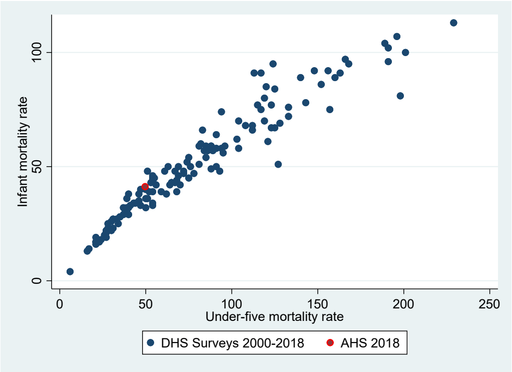

Figure 2. Associations between pregnancy-related mortality rates and early childhood mortality rates in AHS 2018 and DHS surveys conducted between 2000 and 2018 (N=88)

(a) Pregnancy-related and neonatal mortality

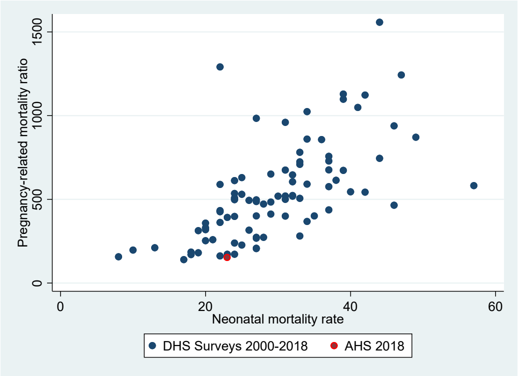

(b) Pregnancy-related and infant mortality

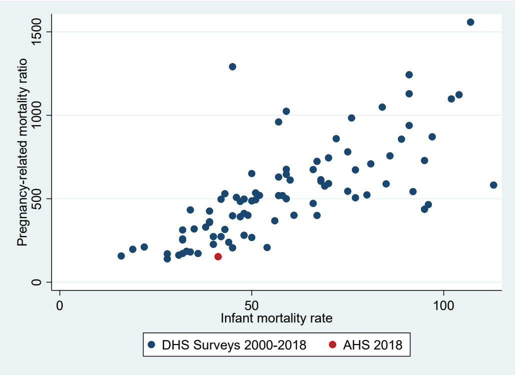

(a) Pregnancy-related and under-five mortality

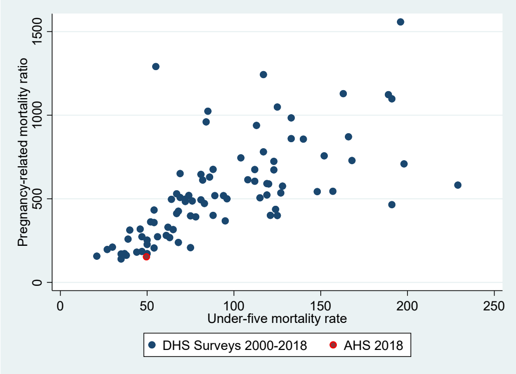

Figure 3. Associations between pregnancy-related mortality rates and maternal health indicators in AHS 2018 and DHS surveys conducted between 2000 and 2018 (N=86)

(a) Pregnancy-related mortality and skilled birth attendance

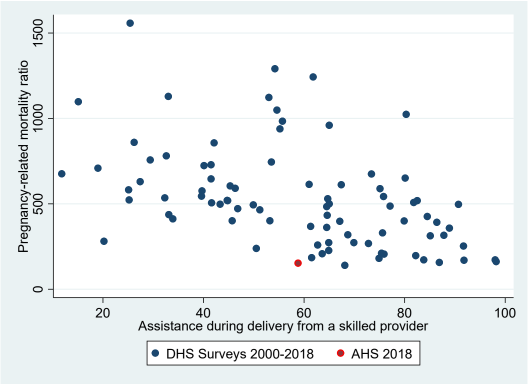

(b) Pregnancy-related mortality and modern contraceptive prevalence

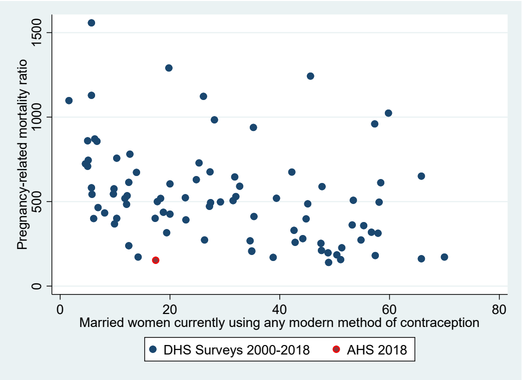

(c) Pregnancy-related mortality and antenatal care

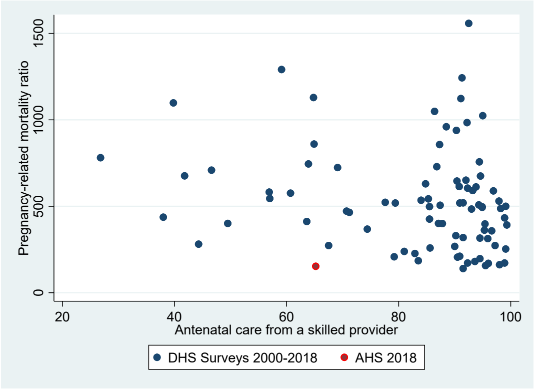

Supplement: Supplementary data [file bmjgh-2019-002126supp001.pdf]
